# The Effect of a 12-Week Omega-3 Supplementation on Body Composition, Muscle Strength and Physical Performance in Elderly Individuals with Decreased Muscle Mass

**DOI:** 10.3390/ijerph120910558

**Published:** 2015-08-28

**Authors:** Roma Krzymińska-Siemaszko, Natasza Czepulis, Marta Lewandowicz, Ewa Zasadzka, Aleksandra Suwalska, Janusz Witowski, Katarzyna Wieczorowska-Tobis

**Affiliations:** 1 Department of Palliative Medicine, Poznan University of Medical Sciences, Poznan 61-245, Poland; E-Mail: kwt@tobis.pl; 2 Department of Pathophysiology, Poznan University of Medical Sciences, Poznan 60-806, Poland; E-Mails: czepulis@ump.edu.pl (N.C.); jwitow@ump.edu.pl (J.W.); 3 Department of Human Nutrition and Hygiene, Poznan University of Life Sciences, Poznan 60-624, Poland; E-Mail: dietetyk.martalewandowicz@gmail.com; 4 Department of Geriatric and Gerontology, Poznan University of Medical Sciences, Poznan 60-781, Poland; E-Mail: ezasad@ump.edu.pl; 5 Department of Adult Psychiatry, Poznan University of Medical Sciences, Poznan 60-572, Poland; E-Mail: asuwalska@gmail.com

**Keywords:** omega-3, sarcopenia, aging, intervention

## Abstract

The aim of the study was to assess the effect of a polyunsaturated omega-3 fatty acids (PUFA) supplementation on the parameters of body composition, muscle strength and physical performance in elderly people with decreased muscle mass (DMM). Fifty three elderly people with an ALM index (the ratio of appendicular lean mass to squared height) either below (−2SD: low muscle mass-LMM) or between (−1SD and −2SD: the risk of LMM-rLMM) the ALM index for the young Polish reference population were randomly assigned to PUFA-treated groups (LMM-PUFA, rLMM-PUFA) or control groups (LMM-control, rLMM-control). PUFA-treated groups received capsules containing 1.3 g of PUFA and 10 mg of vitamin E, while the control groups received 11 mg of vitamin E daily for 12 weeks. Body composition (BIA analysis), muscle strength (hand grip measured with dynamometer) and physical performance (Timed Up and Go test-TUG) were assessed before and after supplementation. No statistically significant differences were observed either in muscle mass or in the hand grip and TUG in any group. The post-pre difference (mean ± SD) in ALM index was as follows (kg/m^2^): LMM-PUFA: 0.00 ± 0.30, rLMM-PUFA: 0.00 ± 0.22, LMM-control: 0.03 ± 0.36, rLMM-control: –0.03 ± 0.20. In our study, a 12 week supplementation of PUFA did not affect the evaluated parameters in elderly individuals with DMM.

## 1. Introduction

Demographic projections show that the number of elderly individuals is continuing to grow. According to the Eurostat data, in 2012, those aged 65 and more represented 17.9% of the European population and 5.1% were at least 80 years old. The elderly population is expected to grow to 28% of the European population by 2050 and the number of those aged 80 or more is predicted to double [1]. Thus, the priority is to ensure that they remain independent.

Human body composition changes over the lifetime. Ageing results in a gradual decline of muscle mass (after the age of 50, at a rate of 1%–2% annually), most frequently accompanied by decreased muscle strength (of 1.5% per year, accelerating to 3% yearly after the age of 60) and/or physical performance deterioration. All this poses a threat to the functional independence of elderly individuals [2,3] due to, for example, an increased risk of falls and decreased ability to perform daily activities, which negatively affect their quality of life [4]. In turn, these processes raise the risk of comorbidity, institutionalization or even death [5].

The reasons for age-related muscle mass decline have only been partly determined. They include hormonal changes, oxidative damage, chronic inflammation, neurodegenerative changes, drugs taken or the presence of certain diseases. Other important factors which are considered to contribute to muscle mass loss include a lifestyle characterized by low physical activity or even lack of mobility due to illness-related immobilization and an unbalanced diet, containing inadequate amounts of calories, lower protein intake than the recommended dietary allowance (RDA= 0.8 g per kg body weight per day both for men and women) or vitamin D deficiency [6,7,8].

Therapeutic agents that might be useful in the prevention and treatment of age-related muscle loss are currently being investigated. Nutritional supplements which may positively influence muscle metabolism appear to be an interesting solution. Some evidence suggests that polyunsaturated omega-3 fatty acids (PUFA) might help preserve muscle mass and function [9,10,11]. In a recent randomized controlled trial, conducted by Smith *et al.* [12], supplementation of older adults’ diets with eicosapentaenoic and docosahexaenoic acids resulted in an enhanced anabolic response to amino acid and insulin infusion and consequently increased muscle protein synthesis.

In the available literature, the effects of omega-3 supplementation on the parameters of body composition and/or muscle strength and physical fitness are mainly evaluated in healthy subjects [13,14]. Omega-3 supplementation is commonly evaluated simultaneously with exposure to physical exercise [15,16]. To the best of our knowledge, the results of the study dedicated to subjects with decreased muscle mass have never been published. It can be hypothesized that an increased intake of omega-3 in these subjects might enhance the synthesis of muscle proteins. The primary objective of the study was to examine the effect of omega-3 supplementation on body composition, muscle strength and physical performance in subjects with decreased muscle mass.

## 2. Material

Subjects included to the study (n = 53) gave their informed written consent for inclusion before they participated in the study. The study was conducted in accordance with the Declaration of Helsinki, and the protocol was approved by the Bioethics Committee of the Poznan University of Medical Sciences, Poland.

### Study Population

Muscle mass was evaluated in 735 community-dwelling elderly individuals aged 60 or above. 158 of them were selected to take part in the study based on their bioimpedance analysis (BIA) evaluated muscle mass results. Only 53 subjects (mean age: 74.6 ± 8.0 years) accepted the invitation to participate in the study. They were recruited in senior clubs in the city of Poznan, the fifth largest city in Poland. 27 participants had low muscle mass (11 men and 16 women) and 26 had a risk of low muscle mass (six men and 20 women). All participants were asked to not change their current nutritional habits and physical activity in the course of the study. No one reported any changes in the pharmacotherapy during the study period.

Inclusion criteria were as follows:

- low muscle mass or the risk of occurrence,
- lack of cognitive function disorders,
- ability to keep a vertical position, which was required for the performance of the body composition test by means of the bioimpedance analysis, due to the type of analyser used.

Exclusion criteria were as follows:

- intake of any steroids,
- intake of anti-platelet and anti-thrombotic drugs, due to the anticoagulant activity of omega-3 fatty acids,
- intake of polyunsaturated omega-3 fatty acids supplements during the 3 months previous to the study,
- presence of metal implants, such as a knee or hip endoprosthesis, or a pacemaker, which is a contraindication for a study conducted by means of bioimpedance analysis.

**Figure 1:**
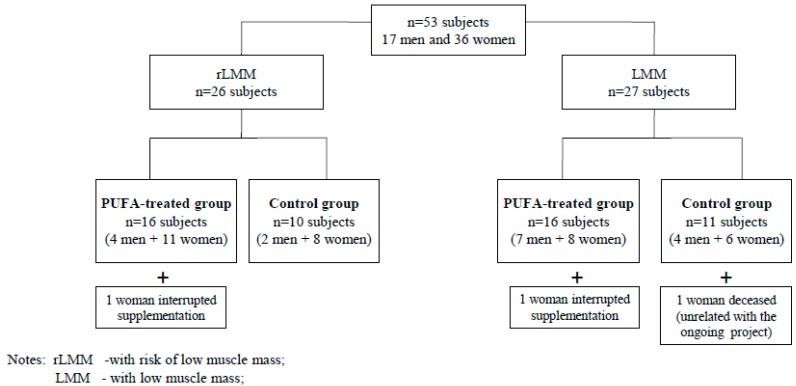
The algorithm of allocation of study subjects to analyzed groups.

The participants were randomly divided into PUFA-treated groups or control groups with low muscle mass (LMM) or a risk of low muscle mass (rLMM) by means of a randomisation list according to Figure 1. The study was not blinded. The subjects from the PUFA-treated groups received 1.3 g of n-3 PUFA over 12 weeks. They took two capsules daily containing 660 mg EPA (eicosapentaenoic acid), 440 mg DHA (docosahexaenoic acid) + 200 mg other omega-3 fatty acids + 10 mg of vitamin E during or immediately after meals.

The subjects from the control groups received 1 drop of vitamin E solution (11 mg) daily, over 12 weeks. Supplements were delivered in monthly intervals. Both the PUFA and vitamin E used in the study are registered and authorised for consumption in Poland by the Chief Sanitary Inspectorate which approves the usage of supplements in Poland. Out of 53 subjects who accepted the invitation to the study only 50 completed it. Two women interrupted their supplementation (due to gastro-intestinal upset) and 1 person died during the observation as a result of an accident unrelated to the study.

## 3. Method

Initially, body composition was assessed through bioimpedance analysis (BIA) and subjects with decreased muscle mass were selected based on muscle mass (see Section 3.1., below). Subsequently, screening for cognitive impairment was performed on all participants. In the case of normal cognitive functions, the nutritional status and independence in activity of daily living were assessed as well as muscle strength and physical performance. The analysis of body composition as well as muscle strength and physical performance were repeated after the 12 week study period. The details of the performed procedures are presented below.

### 3.1. Assessment of Body Composition

Body composition was assessed by means of BIA (InBody 170, Biospace, Seoul, South Korea). The InBody 170 is a segmental impedance device which uses a tetrapolar 8-point tactile electrode method. The device has built-in hand and foot electrodes. Ten impedance measurements are performed using two different frequencies (20 and 100 kHz) at each segment (right arm, left arm, trunk, right leg, and left leg). The subjects wore normal indoor clothes and were advised to stand barefoot in an upright position with their feet placed on the foot electrodes, on the machine platform, and their arms abducted, with hands gripping on to the hand electrodes on the handles. The subject’s identification number, age, sex and height were entered into the analyser. The analyser gives immediate and detailed results, including quantitative values of weight, BMI, skeletal muscle mass, segmental lean mass, fat mass and percentage fat mass. It also calculates appendicular lean mass (ALM) for each subject as the sum of the lean mass of all four limbs. The coefficient of variation for repeated measurements was 0.6% for ALM.

Thereafter, the ALM index (the ratio of ALM (kg) and squared height (m^2^)) was calculated for each subject. Height assessment was performed by means of a mobile stadiometer (Tanita, Poznan, Poland). The ALM index of each elderly individual was compared with the mean ALM index obtained for a young, healthy reference Polish population [17]. Participants were compared separately for each sex. The details of the measurements of the reference population are presented elsewhere [17].

The subjects with low muscle mass (LMM) were selected if their ALM index was not higher than the value obtained by subtracting two standard deviations (−2SD) from the mean ALM index of the reference Polish population, calculated separately for both sexes (5.52 kg/m^2^ for women and 7.29 kg/m^2^ for men [17]). The subjects with a risk of low muscle mass (rLMM) were identified if their ALM index was higher than that in subjects with LMM but lower than the value obtained by subtracting one standard deviation (−1SD) from the mean ALM index of the reference Polish population calculated separately for box sexes (women: not higher than 5.96 kg/m^2^, men: not higher than 7.98 kg/m^2^ [17]). This approach is based on Janssen *et al.*’s [18] criteria for low muscle mass.

### 3.2. Assessment of Cognitive Function

Cognitive functions were assessed with an Abbreviated Mental Test Score (AMTS) [19]. The test is composed of 10 questions. A subject scores 1 point for a correct answer and 0 points for an incorrect answer or no answer. Individuals who score 8 or more are considered cognitively intact. In our study, the score of all the participants qualified for the 12-week observation was within the range of 8–10 points.

### 3.3. Nutritional Assessment

In order to evaluate the nutritional condition of the participants and draw the group characteristics prior to the intervention, the Mini Nutritional Assessment (MNA) was used [20]. MNA is composed of two parts: the so-called Short Form (MNA-SF) and its full version (the full MNA). The MNA-SF contains 6 questions related to the decrease of food intake during the three months prior to the test, body mass loss during this period, subject’s mobility and its limitations, the presence of an acute illness or stress during the preceding three months, as well as of any neuropsychological disorders (depression or dementia). The last question refers to body mass index (BMI).

The full MNA contains 12 additional questions concerning a person’s diet (number of meals consumed, protein, fruit and vegetables and liquid consumption) as well as the ability to eat on one’s own. There are also questions about a self-assessment of the nutritional and health condition, in comparison to other individuals of the same age. Finally, it contains anthropometric measures (arm and calf circumference), as well as information about the number of drugs taken and the presence of bedsores/ulcerations. The maximal score of the full MNA is 30 points. A score below 17 points indicates malnutrition, 17–23.5 points—risk of malnutrition and 24 points or more—normal nutritional status.

### 3.4. Assessment of Independence in Activities of Daily Living

Independence in basic and instrumental activities of daily living was measured by means of the Katz scale and Lawton scale, respectively [21,22]. The Katz scale is composed of six tasks: bathing, dressing and undressing, toileting, transferring from and to bed and continence (bowel and bladder).

A subject scores 1 point for each activity he or she is able to perform independently, 0.5 point if assistance or limited help is needed and 0 points if the person is unable to perform the activity on their own. A total score of 0–2 points indicates an impairment of daily living performance, 3–4 points: a moderate impairment and a score of 5–6 points stands for complete independence.

The Lawton scale assesses performance in nine dimensions: ability to use telephone, ability to use different modes of transportation, shopping, food preparation, housekeeping (doing laundry and cleaning), performing simple repair tasks, control over one’s own medications and the ability to handle finances. A subject can score 0–3 points for each activity. 3 points stand for complete independence, 2 points indicate that a limited help is needed and 1 point means that the person is not able to perform the activity. The maximum score is 27 points. As far as the Lawton scale is concerned, there are no cut-off points that would define different levels of independence. However, it does allow for profiling a patient’s needs in terms of assistance or care as lower results indicate a higher dependence level.

### 3.5. Assessment of the Muscle Strength and Physical Performance

Muscle strength was assessed by hand grip strength with a dynamometer (Saehan, Changwon, South Korea). Participants performed the hand grip strength test in the standing position with full elbow extension and 90 degrees of shoulder flexion. Both the left and right arms were tested once. The mean value of both hands was used as the final score for each individual. The results were recorded in kilograms (kg). The coefficient of variation for repeated hand grip measurements was 6.0%.

Physical performance was assessed by means of the Timed Up and Go Test (TUG), as well as the 4-meter walking test. A stopwatch (Stratos 2 Hanhart, Oakland, NJ USA) was used to time both tests (in seconds). The coefficients of variation for repeated TUG and 4-meter Walking Test measurements were 2.0% and 5.3%, respectively.

TUG consists of the following tasks: transfer from sitting to standing position, a fast, short-distance walk on a flat surface (3 m), and returning to the sitting position. If a subject walks with a cane or crutch(es), he or she performs the test using the necessary aid(s). The time needed to complete the test above 14 s represents an increased risk of falls [23,24].

The 4-meter Walking Test measures the walking pace at the distance of 4 metres. Subjects have to walk a 4-meter course at their usual gait speed. Time taken to perform the walk was recorded and expressed as meters per second. The walking speed below 0.8m/s indicates a risk of functional dependence [25].

### 3.6. Statistical Analysis

Statistical analysis was conducted by means of the STATISTICA 10.0 software (StatSoft, Cracow, Poland). Mean values and standard deviations were calculated for the parameters analysed. Normality in the distribution of variables was assessed with the Shapiro-Wilk test. Due to lack of normality non parametric two-way ANOVA was used to evaluate before-to-after intervention changes in all studied groups. The alpha level for the study was set at 0.05. In our study we did not implement the power tests because we decided to include all people who meet inclusion criteria regardless of their number.

## 4. Results

Fifty subjects (33 women and 17 men) completed the 12-week observation. Thirty participants took omega-3 fatty acids (the PUFA-treated group), while the remainder of the group were administered vitamin E (the control group). Figure 1 presents the algorithm of allocation of study subjects to analysed groups.

The age and sex of both groups were comparable (see Table 1). At the beginning of the study the groups did not differ by any other studied parameters (*i.e.*, Abbreviated Mental Test Score, Katz’ index, Lawton scale and Mini Nutritional Assessment). Both groups included individuals who were independent in terms of their basic and instrumental activities of daily living and whose nutritional status was good.

The studied parameters, separately for the participants with low muscle mass (LMM) and participants exposed to such risk (rLMM) are presented in detail in Table 2 and Table 3, respectively. All studied subjects were independent in good condition. The data of body composition, muscle strength and physical performance prior to and following the 12-week observation in all studied subjects in PUFA-treated groups (n = 30) and control groups (n = 20) are presented in Table 4. Table 5 and Table 6 contain above parameters respectively for individuals with low muscle mass (n = 25; n = 15 LMM-PUFA-treated group and n = 10 LMM-control group) and its risk (n = 25; n = 15 rLMM-PUFA-treated group and n = 10 rLMM-control group). No statistically significant differences were observed in the analysed components of body composition in any group (see Table 4, Table 5 and Table 6). It must be emphasised that after the 12-week observation the changes in muscle mass assessed by the ALM index were comparable in all groups.

**Table 1:** The characteristics of studied subjects of the PUFA-treated and controls groups.

Variable	PUFA-Treated Groups: LMM + rLMM	Control Groups: LMM + rLMM	*p*
Sex			
Women (n)	19	14	
Men (n)	11	6	
Age (years)	74.97 ± 8.23	74.85 ± 7.49	0.96
AMTS (points)	9.00 ± 0.80 *	8.95 ± 0.92 **	0.88
Katz’ index (points)	5.92 ± 0.30	5.70 ± 1.12	0.59
Lawton scale (points)	25.93 ± 2.18	24.45 ± 4.38	0.17
MNA (points)	24.76 ± 2.89 *	23.39 ± 3.25 **	0.16

Notes: the table includes the results of those individuals who finish the 12-weeks intervention only (n = 50); * n = 29; ** n = 19; Abbreviations: AMTS, Abbreviated Mental Test Score; MNA, Mini Nutritional Assessment.

**Table 2:** The characteristics of studied subjects with low muscle mass (LMM) only.

Variable	LMM PUFA-Treated Group	LMM Control Group	*p*
Sex			
Women (n)	8	6	
Men (n)	7	4	
Age (years)	74.87 ± 8.99	77.20 ± 6.81	0.49
AMTS (points)	9.14 ± 0.95 *	9.11 ± 0.93 **	0.92
Katz’ index (points)	5.90 ± 0.39	5.45 ± 1.57	0.35
Lawton scale (points)	25.33 ± 2.89	22.50 ± 5.50	0.08
MNA (points)	24.53 ± 2.77	21.60 ± 2.98	0.02

Notes: the table includes the results of those individuals who finish the 12-weeks intervention only (n = 25); * n = 14; ** n = 9; Abbreviations: AMTS, Abbreviated Mental Test Score; MNA, Mini Nutritional Assessment.

All the studied parameters with regard to muscle strength and physical performance were also comparable in all the studied groups. The average muscle strength did not change after 12-week supplementation with PUFA neither in LMM-PUFA nor in rLMM-PUFA similarly to controls. Also the pre-post difference for both 4-meter Walking Test and Timed Up and Go Test were comparable in PUFA-treated groups and in controls.

**Table 3:** The characteristics of studied subjects with risk of low muscle mass (rLMM) only.

Variable	rLMM PUFA-Treated Group	rLMM Control Group	*p*
Sex			
Women (n)	11	8	
Men (n)	4	2	
Age (years)	75.07 ± 7.71	72.50 ± 7.74	0.42
AMTS (points)	8.87 ± 0.64	8.80 ± 0.92	0.98
Katz’ index (points)	5.93 ± 0.18	5.95 ± 0.16	0.84
Lawton scale (points)	26.53 ± 0.83	26.40 ± 1.35	0.88
MNA (points)	25.00 ± 3.10	25.39 ± 2.29	0.75

Notes: the table includes the results of those individuals who finish the 12-weeks intervention only (n = 25); Abbreviations: AMTS, Abbreviated Mental Test Score; MNA, Mini Nutritional Assessment.

## 5. Discussion

A number of studies have been carried out on the influence of PUFA on body composition, particularly on muscle mass and lean body mass in healthy individuals. After a 6-week omega-3 supplementation (1.6 g EPA + 0.8 g DHA daily) in young, healthy individuals (n = 24, mean age: 33.0 ± 13.0), Noreen *et al.* [13] have shown that it resulted in an increase of lean body mass. Moreover, Smith *et al.* [12], who studied eight healthy, but inactive, elderly individuals (mean age: 71.0 ± 1.0), reported—after an 8-week supplementation (1.86g EPA and 1.5g DHA daily)—that it resulted in the improvement of muscle protein synthesis. Importantly, however, the size of the studied groups described in the above papers was small.

On the other hand, Couet *et al.* [14] have not observed a significant increase in lean body mass in young, healthy individuals (n = 6, mean age: 23.0 ± 2.0) following a 3-week supplementation despite a very high dose of omega-3 fatty acids e.g., 6 g daily. Here again the studied group was very small. Moreover, Welch *et al.* [26] even found an inverse relationship between dietary intakes of omega-3 and lean body mass in a cohort study on 1354 women aged 50 and older, with normal muscle mass.

**Table 4:** Body composition, muscle strength and physical performance before and after 12-week intervention period in all studied subjects (with low muscle mass—LMM and risk of low muscle mass—rLMM).

Variable	PUFA-Treated Groups: LMM + rLMM			Control Groups: LMM + rLMM			
	Pre	Post	Post-Pre Difference	Pre	Post	Post-Pre Difference	*p* *
Sex							
Women (n)	19	19		14	14		
Men (n)	11	11		6	6		
Age (years)	74.97 ± 8.23			74.85 ± 7.49			
Height (m)	1.57 ± 0.08			1.59 ± 0.09			
Weight (kg)	57.56 ± 8.43	57.66 ± 8.94	0.10 ± 1.71	57.96 ± 11.50	58.33 ± 11.68	0.37 ± 1.68	0.99
Body Mass Index (kg/m^2^)	23.41 ± 3.14	23.44 ± 3.36	0.03 ± 0.72	22.93 ± 3.39	23.06 ± 3.38	0.13 ± 0.66	0.97
Skeletal Muscle Mass (kg)	21.46 ± 3.89	21.54 ± 4.17	0.08 ± 1.09	21.41 ± 4.19	21.47 ± 4.62	0.06 ± 1.00	0.99
Fat (kg)	17.20 ± 6.79	17.48 ± 7.36	0.28 ± 1.64	17.75 ± 6.44	18.08 ± 6.52	0.33 ± 2.03	0.94
Percent Body Fat (%)	29.48 ± 9.08	29.81 ± 9.47	0.33 ± 2.33	30.11 ± 6.74	30.60 ± 7.25	0.49 ± 2.87	0.92
Total Body Water (l)	29.68 ± 5.09	29.53 ± 5.27	−0.15 ± 1.15	29.56 ± 5.27	29.58 ± 5.84	0.02 ± 1.29	0.98
Fat-Free Mass (kg)	40.36 ± 6.78	40.19 ± 7.06	−0.18 ± 1.55	40.21 ± 7.10	40.25 ± 7.91	0.04 ± 1.78	0.98
Segmental Lean-Right Arm (kg)	2.11 ± 0.53	2.09 ± 0.57	−0.01 ± 0.16	2.01 ± 0.57	2.03 ± 0.58	0.02 ± 0.11	0.98
Segmental Lean-Left Arm (kg)	2.08 ± 0.54	2.06 ± 0.57	−0.02 ± 0.17	1.97 ± 0.55	2.02 ± 0.59	0.05 ± 0.12	0.95
Segmental Lean-Right Leg (kg)	5.62 ± 1.29	5.64 ± 1.26	0.02 ± 0.20	5.68 ± 1.40	5.62 ± 1.43	−0.05 ± 0.29	0.98
Segmental Lean-Left Leg(kg)	5.63 ± 1.24	5.63 ± 1.21	0.00 ± 0.20	5.67 ± 1.28	5.65 ± 1.37	−0.02 ± 0.32	0.99
Appendicular Lean Mass (kg)	15.43 ± 3.48	15.42 ± 3.49	−0.01 ± 0.63	15.33 ± 3.66	15.33 ± 3.86	0.00 ± 0.72	0.99
ALM index (kg/m^2^)	6.19 ± 0.82	6.19 ± 0.82	0.00 ± 0.26	6.01 ± 0.92	6.01 ± 1.00	0.00 ± 0.29	0.80
Average Muscle Strength (kg)	25.70 ± 7.20	25.53 ± 7.18	−0.17 ± 1.91	21.66 ± 8.16 **	22.51 ± 7.12 **	0.85 ± 3.14 **	0.17
Timed Up and Go Test (s)	6.97 ± 2.66 **	7.15 ± 1.86 *	0.17 ± 1.17 *	7.82 ± 2.66 **	7.95 ± 2.84 **	0.13 ± 1.13 **	0.62
4-meter Walking Test (s)	1.43 ± 0.37 ***	1.57 ± 0.47 ***	0.14 ± 0.39 ***	1.32 ± 0.38 **	1.51 ± 0.59 **	0.19 ± 0.49 **	0.32

Notes: * n = 26; ** n = 19; *** n = 29 ; missing data are due to inclusion of subjects who were not able to follow the instruction of Timed Up and Go Test i 4-meter Walking Test; Abbreviations**:** ALM index, Appendicular Lean Mass index.

**Table 5:** Body composition, muscle strength and physical performance before and after 12-week intervention period in subjects with low muscle mass (LMM) only.

Variable	LMM PUFA-Treated Group			LMM Control Group			p *
	Pre	Post	Post-Pre Difference	Pre	Post	Post-Pre Difference	
Sex							
Women (n)	8	8		6	6		
Men (n)	7	7		4	4		
Age (years)	74.87 ± 8.99			77.20 ± 6.81			
Height (m)	1.57 ± 0.07			1.58 ± 0.10			
Weight (kg)	57.23 ± 9.41	57.07 ± 9.81	–0.17 ± 1.54	55.52 ± 12.47	56.03 ± 12.33	0.51 ± 1.66	0.83
Body Mass Index (kg/m^2^)	23.08 ± 3.30	22.97 ± 3.33	–0.11 ± 0.64	22.10 ± 3.59	22.30 ± 3.54	0.20 ± 0.67	0.69
Skeletal Muscle Mass (kg)	21.74 ± 3.99	21.50 ± 4.18	–0.24 ± 0.96	20.70 ± 4.48	20.86 ± 5.13	0.16 ± 1.22	0.80
Fat (kg)	16.63 ± 6.15	16.99 ± 6.50	0.35 ± 0.99	16.32 ± 6.60	16.58 ± 6.02	0.26 ± 2.46	0.95
Percent Body Fat (%)	28.72 ± 7.43	29.39 ± 7.70	0.67 ± 1.80	28.84 ± 6.97	29.39 ± 6.84	0.55 ± 3.54	0.98
Total Body Water (l)	29.85 ± 5.10	29.47 ± 5.32	–0.38 ± 1.20	28.83 ± 5.61	29.00 ± 6.51	0.17 ± 1.62	0.93
Fat-Free Mass (kg)	40.60 ± 6.77	40.08 ± 7.09	–0.52 ± 1.60	39.20 ± 7.60	39.45 ± 8.84	0.25 ± 2.22	0.89
Segmental Lean-Right Arm (kg)	2.15 ± 0.58	2.15 ± 0.62	–0.01 ± 0.18	1.90 ± 0.57	1.93 ± 0.61	0.03 ± 0.12	0.49
Segmental Lean-Left Arm (kg)	2.13 ± 0.57	2.12 ± 0.60	–0.01 ± 0.20	1.87 ± 0.53	1.91 ± 0.58	0.04 ± 0.15	0.49
Segmental Lean-Right Leg (kg)	5.54 ± 1.12	5.55 ± 1.12	0.02 ± 0.23	5.46 ± 1.51	5.44 ± 1.64	–0.02 ± 0.35	0.94
Segmental Lean-Left Leg(kg)	5.51 ± 1.13	5.52 ± 1.13	0.01 ± 0.22	5.48 ± 1.42	5.49 ± 1.59	0.02 ± 0.42	0.98
Appendicular Lean Mass (kg)	15.33 ± 3.32	15.33 ± 3.41	0.01 ± 0.75	14.70 ± 3.95	14.77 ± 4.35	0.07 ± 0.92	0.88
ALM index (kg/m^2^)	6.12 ± 0.89	6.12 ± 0.90	0.00 ± 0.30	5.77 ± 0.98	5.80 ± 1.14	0.03 ± 0.36	0.53
Average Muscle Strength (kg)	26.19 ± 7.13	26.87 ± 6.65	0.68 ± 1.43	21.09 ± 7.70 **	21.63 ± 6.41 **	0.54 ± 2.77 **	0.12
Timed Up and Go Test (s)	7.06 ± 3.51 *	7.11 ± 2.24 *	0.05 ± 1.50 *	8.93 ± 3.12 **	9.35 ± 3.46 **	0.42 ± 1.18 **	0.11
4-meter Walking Test (s)	1.46 ± 0.42 ***	1.57 ± 0.44 ***	0.11 ± 0.26 ***	1.16 ± 0.27 **	1.25 ± 0.23 **	0.09 ± 0.13 **	0.06

Notes: * n = 13; ** n = 9; *** n = 14; missing data are due to inclusion of subjects who were not able to follow the instruction of Timed Up and Go Test i 4-meter Walking Test; Abbreviations: ALM index, Appendicular Lean Mass index.

**Table 6:** Body composition, muscle strength and physical performance before and after 12-week intervention period in subjects with risk of low muscle mass (rLMM) only.

Variable	rLMM PUFA-Treated Group			rLMM Control Group			*p* *
	Pre	Post	Post-Pre Difference	Pre	Post	Post-Pre Difference	
Sex							
Women (n)	11	11		8	8		
Men (n)	4	4		2	2		
Age (years)	75.07 ± 7.71			72.50 ± 7.74			
Height (m)	1.56 ± 0.09			1.59 ± 0.08			
Weight (kg)	57.89 ± 7.65	58.26 ± 8.29	0.37 ± 1.88	60.39 ± 10.51	60.62 ± 11.14	0.23 ± 1.79	0.93
Body Mass Index (kg/m^2^)	23.74 ± 3.05	23.92 ± 3.43	0.17 ± 0.80	23.76 ± 3.15	23.83 ± 3.22	0.06 ± 0.68	0.97
Skeletal Muscle Mass (kg)	21.17 ± 3.89	21.57 ± 4.31	0.40 ± 1.14	22.12 ± 3.99	22.07 ± 4.24	−0.05 ± 0.77	0.55
Fat (kg)	17.76 ± 7.55	17.97 ± 8.33	0.21 ± 2.14	19.17 ± 6.28	19.57 ± 6.97	0.40 ± 1.62	0.83
Percent Body Fat (%)	30.24 ± 10.70	30.24 ± 11.23	0.00 ± 2.79	31.37 ± 6.63	31.80 ± 7.80	0.43 ± 2.19	0.92
Total Body Water (l)	29.51 ± 5.25	29.59 ± 5.41	0.09 ± 1.10	30.28 ± 5.09	30.15 ± 5.38	−0.13 ± 0.92	0.62
Fat-Free Mass (kg)	40.13 ± 7.01	40.29 ± 7.28	0.17 ± 1.47	41.22 ± 6.81	41.05 ± 7.25	−0.17 ± 1.29	0.64
Segmental Lean-Right Arm (kg)	2.06 ± 0.49	2.04 ± 0.53	−0.02 ± 0.13	2.13 ± 0.58	2.13 ± 0.56	0.00 ± 0.11	0.82
Segmental Lean-Left Arm (kg)	2.03 ± 0.53	2.00 ± 0.55	−0.03 ± 0.14	2.08 ± 0.58	2.13 ± 0.62	0.05 ± 0.10	0.66
Segmental Lean-Right Leg (kg)	5.70 ± 1.48	5.73 ± 1.43	0.03 ± 0.18	5.89 ± 1.32	5.81 ± 1.24	−0.08 ± 0.22	0.82
Segmental Lean-Left Leg(kg)	5.75 ± 1.37	5.74 ± 1.32	−0.01 ± 0.18	5.87 ± 1.15	5.81 ± 1.17	−0.06 ± 0.19	0.87
Appendicular Lean Mass (kg)	15.54 ± 3.75	15.51 ± 3.68	−0.03 ± 0.53	15.97 ± 3.45	15.89 ± 3.46	−0.08 ± 0.50	0.72
ALM index (kg/m^2^)	6.26 ± 0.76	6.26 ± 0.76	0.00 ± 0.22	6.25 ± 0.84	6.21 ± 0.85	−0.03 ± 0.20	0.98
Average Muscle Strength (kg)	25.21 ± 7.50	24.18 ± 7.66	−1.03 ± 1.99	22.18 ± 8.94	23.30 ± 7.96	1.12 ± 3.56	0.80
Timed Up and Go Test (s)	6.90 ± 1.79 *	7.18 ± 1.55 *	0.28 ± 0.84 *	6.83 ± 1.77	6.69 ± 1.35	−0.14 ± 1.07	0.76
4-meter Walking Test (s)	1.40 ± 0.33	1.58 ± 0.51	0.18 ± 0.49	1.47 ± 0.41	1.74 ± 0.72	0.28 ± 0.66	0.76

Notes: * n = 14; missing data are due to inclusion of the subject who was not able to follow the instruction of Timed Up and Go Test i 4-meter Walking Test; Abbreviations: ALM index, Appendicular Lean Mass index.

In our study, the 12-week supplementation with omega-3 did not significantly affect muscle strength. In agreement with our study, Hutchins-Wiese *et al.* [33] have shown—in 85 elderly women (mean age: 75.0 ± 6.0) who were pre-frail or frail—that supplementation with 1.2 g of omega-3 (0.72 g EPA and 0.48 g DHA) for six months was ineffective in terms of muscle strength. The dose of PUFA used in that study was similar to the one used by us but the intervention period was twice longer. As far as the physical performance is concerned, they found that PUFA had a beneficial effect on walking speed but not on results of a repeated chair rise test. Thus, it seems possible that a walking speed test is more sensitive than chair rise test [34]. However, in our study the walking speed did not change after the 12-weeks of intervention. This may be due to the fact that the intervention was too short.

Rodacki *et al.* [15] have found, however, that when healthy elderly females (n = 15; mean age: 63.3 ± 2.0 years) were treated with PUFA (1.2 g EPA and 0.9 g DHA) for 60 days, the intervention was not effective. This is in agreement with our results. However, our results also showed that when supplementation was performed (n = 15, mean age: 64.9 ± 1.0 years) for 60 days, simultaneously with strength training, a greater improvement was observed in comparison with strength training only (n = 15, mean age: 63.8 ± 1.4). In addition, Cornish and Chilibeck [16] have found that the addition of 14 g ALA per day in older adults (n = 51; mean age: 65.4 ± 0.8 years) who were exposed to resistance training (three days a week during 12 weeks) resulted in only minimal improvement of lean tissue mass and muscle strength in comparison with resistance training alone.

It seems possible that simultaneous supplementation with PUFA and strength training is required in order to achieve a beneficial effect. This has already been suggested by Sugawara *et al.* [35], who postulated that PUFA boosted the effect of enhanced physical activity. However, further studies are needed to prove this hypothesis.

### Study Limitations

Our study has certain limitations. Firstly, we used the BIA method to assess the changes in muscle mass. The DEXA method is a preferred alternative for research and clinical use. It is, however, not feasible to measure muscle mass in community-dwelling elderly adults with DEXA. BIA is thus a more practical screening method to be used in large samples, especially in a community setting. Moreover, there are more and more papers attesting a high level of agreement of the newest generation BIA devices (*i.e.*, those using the 8-point tactile electrode method with more than 1 frequency—as was used by us) with the DEXA method [36,37,38].

Secondly, a relatively small group of elderly subjects participated in the 12-week supplementation. This resulted from the fact that low muscle mass and its risk were infrequent among the community-dwelling elderly who were participating in activities of the local senior centres. They are potentially much more robust than the average elderly subjects.

Thirdly, a relatively low PUFA dose—1.3 g/d (including 0.66 g EPA and 0.44 g DHA)—was used in our study in comparison with the other studies. However, we based our dosage on the Polish recommendations on omega-3 fatty acids intake: 1.0 g/d of omega-3 daily was defined as the optimal omega-3 consumption for the Polish population, while the groups with an increased risk of cardiovascular, rheumatoid, cancer and neurodegenerative diseases were stated to need 1.5 g/d [39]. Nevertheless, some positive effects have previously been found with the same dose of PUFA supplementation [33].

Fourthly, the 12-week intervention period might have been too short to generate beneficial effects on muscle mass and functions. A longer observation period is needed owing to the fact that the age-related alterations in the muscles progress gradually over many years. On the other hand, beneficial effects have been found even when the supplementation period was shorter [12,13,14]. It must be pointed out that we supplemented the diet with PUFA. The effect of a diet containing food rich in PUFA may potentially be different.

## 6. Conclusions

The 12-week supplementation with polyunsaturated omega-3 fatty acids did not positively affect body composition, muscle strength or physical performance in elderly individuals with low muscle mass or with the risk of low muscle mass. However, we believe that conclusions should be drawn with caution as the number of observations is relatively small Thus, it is important to note that our results do not exclude the protective role these fatty acids may play for ageing muscles. They also indicate that further studies on larger groups of elderly individuals both with normal and low muscle mass are needed, possibly also with higher doses.
